# Tumor-Derived Extracellular Vesicles and the Immune System—Lessons From Immune-Competent Mouse-Tumor Models

**DOI:** 10.3389/fimmu.2020.606859

**Published:** 2020-12-16

**Authors:** Marvin Droste, Basant K. Thakur, Brian P. Eliceiri

**Affiliations:** ^1^ Department of Surgery, Division of Trauma, Surgical Critical Care and Burns, UC San Diego School of Medicine, San Diego, CA, United States; ^2^ Department of Pediatrics II (Pediatric Nephrology), University Hospital Essen, Essen, Germany; ^3^ Cancer Exosomes Laboratory, Department of Pediatrics III, University Hospital Essen, Essen, Germany

**Keywords:** extracellular vesicles, tumor exosomes, adaptive immunity, extracellular vesicle heterogeneity, cancer, immunotherapy, mouse tumor models, immune system

## Abstract

Tumor-derived extracellular vesicles (TEVs) are important regulators of the immune response in cancer; however, most research so far has been carried out using cell culture systems. Immune-competent murine tumor models currently provide the best platform to assess proposed roles of TEVs using *in vivo* animal models and therefore are important for examining interactions between TEVs and the immune system. In this review, we present the current knowledge on TEVs using *in vivo* tumor-bearing animal models, with a focus on the role of TEVs in mediating crosstalk between tumor cells and both adaptive and innate immune cells. In particular, we address the question how animal models can clarify the reported heterogeneity of TEV effects in both anti-tumor responses and evasion of immune surveillance. The potential of TEVs in mediating direct antigen-presenting functions supports their potential as cancer vaccine therapeutics, therefore, we provide an overview of key findings of TEV trials that have the potential as novel immunotherapies, and shed light on challenges in the path toward the first in-human trials. We also highlight the important updates on the methods that continue to enhance the rigor and reproducibility of EV studies, particularly in functional animal models.

## Introduction

Metastatic cancers are among the deadliest diseases worldwide, yet, therapy options remain limited. Escaping the host’s immune response is one of the characteristic properties of tumors that are essential for malignancy, tumor growth and metastasis (1). During the past few decades, immuno-oncology research has focused on deciphering the molecular mechanisms that tumors develop to influence their microenvironment as well as the global immune response. More recently, extracellular vesicles (EVs) released by tumor cells have been studied extensively for their potential as antigen-presenting particles and regulators of immune cells (2). EVs are nanoparticles that are formed intracellularly, and are composed of a proteo-lipid bilayer, and luminal proteins and nucleic acids, which together determine their functionality. The complex mechanism of EVs biogenesis has been recently outlined elsewhere (3). Of note, the packaging of EVs may be altered upon changes of the cellular microenvironment or after therapeutic interventions (4). EVs are categorized by size and the organelle from which they are derived. The three predominant classes of EVs are endosomal-derived exosomes that are 50–150 nm in diameter, microvesicles, or “ectosomes” that are 200–1,000 nm in diameter and are derived from plasma membrane shedding, and apoptotic bodies that have a wide size range of 100–5,000 nm in diameter. Recent work has pointed toward the existence of a plethora of additional EV classes not described above, a prominent example being so-called exomeres. Exomeres were described to be smaller than exosomes (<50 nm) and enriched in metabolism-associated enzymes and antigens, which has led to the assumption that they might influence the metabolic state of recipient cells and take part in tumor-related mechanisms such as metastasis (5, 6).

The heterogeneity of extracellular vesicles is further underscored by studies that continue to refine our understanding of their cargo (7), and challenges with standardized isolation and characterization techniques to define the function of specific subpopulations of EVs. It remains a common approach in the field to focus studies on pellets acquired following ultracentrifugation. These fractions are referred to as “exosomes”, even though the International Society for Extracellular Vesicles (ISEV) is encouraging researchers to use the term “small EVs” to better capture the spectrum of vesicle types (8). To meet the requirements of more precise terminology and biological accuracy, we will use the term “tumor-derived extracellular vesicles” (TEVs), which includes vesicles released by an endosomal pathway (“classical” exosomes), membrane-derived vesicles and apoptotic-derived vesicles since all three are frequently observed in malignancy.

Many investigators have identified and classified TEVs using various cancer cell lines to obtain an insight into their pathophysiological relevance in tumor progression and immune responses, however, the potential of TEVs as immune regulators in immune-competent animal tumor models remains understudied. The importance of an immune-competent model is highlighted by the need to understand tumor-immune cell-crosstalk mediated by TEVs in the complexity of an animal model, which we argue have the greatest potential to provide insight into the TEV-dependent mechanisms relevant to metastasis and translation to humans. We point out where findings from animal studies and cell culture experiments may appear to be contradictory and identify areas for future research on the role of TEVs and the regulation of tumor-associated immune cells.

An understanding of the basis of the most utilized methods, such as direct injection of TEVs and immune cell pulsing by TEVs before injection, is important to consider with respect to their potential influences on experimental outcomes. Work by Andre and colleagues (9) first showed that TEVs carry antigens derived from their cells of origin that are presented by dendritic cells (DCs) to induce the proliferation of specific T cell subsets. The observation that bodily fluids contain TEVs that are reservoirs for tumor antigens demonstrated the relevance of TEVs in tumor immunotherapy research that has been followed by cancer vaccine trials (10) and TEV bioengineering approaches (11, 12). While specific aspects of TEV immunology remain poorly understood, TEVs remain promising targets for future research, with the regulation of their release being a prominent example (13–16). Moreover, the rapid evolution of EV methodologies in general will benefit *in vivo* studies of TEVs. Progress in these areas will likely lead to new therapy options in cancer, autoimmune disease and many other chronic conditions where EVs can mediate immune activity to distal end organs.

## TEV Effects on the Adaptive Immunity: Dendritic Cells and T Cells

Our current understanding of anti-tumor immunity emphasizes the role of CD8^+^ cytotoxic T-lymphocytes (CTLs) as key protective agents. The presentation of tumor antigens by dendritic cells induces the activation and clonal proliferation of anti-tumor CTLs, however, tumors regularly develop mechanisms and factors to evade this response. It has been recognized that TEVs might carry such factors as a payload or express tumor antigens on their surface. Therefore, both immunosuppressive and immune-stimulatory roles have been proposed and described for TEVs (2).

In terms of the immunosuppressive effects of TEVs, direct as well as indirect effects on cellular immunity have been proposed (17). Ning and colleagues demonstrated in a murine model that TEVs owe the potential to block the differentiation and function of dendritic cells and Th1 CD4^+^ lymphocytes, whereas the activity of regulatory T cells (Tregs) is increased by TEVs (18). However, functional conclusions should be drawn with caution as certain Treg subtypes may also suppress cancer progression especially in tumors with extended tumor-induced inflammation (19).

Although not much is known about the pathways by which TEVs can directly suppress effector cell function, there is some recent evidence that apoptosis induction, at least in CD4^+^ T cells, is mediated by miRNA that is associated with TEVs (20), a mode of EV signaling that has been well established since the recognition of various RNA types as a common EV cargo (21). The immunosuppressive effects of TEVs were partially reversible by blockage of PD-L1, allowing the interpretations that either TEVs carry PD-L1 on their surface to interact with PD-1 receptors on activated CTL, or that TEVs induced the expression of PD-L1 on dendritic cells. It was recently demonstrated that tumor cells actively secrete TEVs carrying PD-L1 that are released in a mechanism that is dependent on the EV release regulators such as Rab27a and nSMase2 (22, 23). In mice, the TEV-associated PD-L1 enhanced tumor progression, suppressed T cell activity, and was resistant to antibody therapy. Interestingly, when TEV-associated PD-L1 secretion was blocked, distant secondary tumor growth was inhibited (23). This effect was synergistic with anti-PD-L1 antibody treatments and led the authors to the conclusion that blocking of PD-L1 secretion associated with TEVs could provide an additive treatment to current antibody therapies, which would have a high clinical relevance (24). Interestingly, PD-L1 has been shown to be present in TEVs from various cancer cell lines in different concentrations, which could explain the heterogeneous results obtained from therapeutic PD-L1 antibody treatment (23). Further studies are warranted to clarify whether other tumor-derived ligands are also differentially packaged into TEVs dependent on the cancer cell line.

Another example for a mechanism of CD4^+^ and CD8^+^ T cell suppression is arginase-1 carried by TEVs. Such TEVs were isolated from patients with ovarian cancer and inhibited effector cell function after previous uptake into dendritic cells in an ovarian cancer mouse model (25). The resulting tumor progression could be reversed by applying arginase inhibitors, thus underlining the potential for therapeutic manipulation.

Despite suppression of T cell function, it has also been reported that TEVs carry out tumor antigen-presenting functions, similar to antigen-presenting cells. When CD8^+^ CTL are stimulated *in vitro* with TEVs, a strong specific anti-tumor effect of these CTL was reported (26). This finding might open up a new strategy for potential tumor vaccines. Additionally, this observation raises the question whether TEVs alone have the capacity to induce T cell responses independently of the contribution by dendritic cells. Indeed, another group has also described a direct antigen-presenting effect of TEVs on CD8^+^ CTL *via* transfer of so-called pioneer translation peptides that originate from pre-mRNAs (27). In contrast, others have shown that TEVs alone do not exert T cell-activating functions in the absence of DCs even though they carry MHC cargo (28). This finding has been attributed to the fact that TEVs, due to their small size, might not carry enough MHC molecules to activate the T cell receptor (29). It remains controversial whether TEVs may act as “mini-DCs” to directly promote T cell activation, which can be addressed in the future by comparison of multiple tumor models. The potential role of vesicle size can be addressed by the use of different TEV isolation protocols.

During the last decade, multiple groups have investigated the processes by which TEVs interact with T cells to promote the effects discussed above. Most evidence which is available to date points toward a receptor-ligand interaction between T cells and TEVs which controls cellular homeostasis. For instance, it has been demonstrated that binding of TEVs to target receptors on T cells alters the amount of Ca^2+^ influx and comes along with transcriptional reprogramming of the targeted cell (30, 31). In this context, the signaling ligands of TEVs may function in the same way as cells carrying the surface molecule (32). However, the proposed transfer of RNA to T cells would require internalization of TEVs and subsequent intracellular cargo release. Indeed, recent work has suggested the existence of a relatively small subpopulation of T cells that is able to internalize TEVs by micropinocytosis, at least in a growth factor-enriched microenvironment such as the tumor bed (33). Given the fact that the variety of TEV uptake mechanisms is thought to be large, other processes, such as receptor-mediated endocytosis, may also play a role, as comprehensively outlined elsewhere (34, 35).

Antigen-presenting cells, such as DCs, are another important target of TEVs, as they largely facilitate the connection between tumor-derived, antigen-containing material, and immune effector cells. TEVs have been described to either induce or decrease the intensity by which dendritic cells are activated, comparable to what is known regarding the TEV effects on T cells. It was reported by several groups that TEVs severely interfere with differentiation of DCs from bone marrow myeloid precursor cells and CD14^+^ monocytes, resulting in impaired migratory behavior and apoptosis of the precursor cells (18, 36). Moreover, when incubated with TEVs, these cells produce increased amounts of interleukin-6 (IL-6) (36). Interestingly, IL-6 induction was also shown in TEV-activated differentiated DCs and one study reported that this is related to metastasis and tumor invasion in a STAT3-dependent manner (37). However, this trial did not provide evidence that this can be attributed to TEVs *in vivo*, as IL-6 induction by TEVs was solely demonstrated *in vitro*. In addition, the effect has been linked to HSP72 on the TEVs, which conversely has been shown by another group to induce IL-12 production and thus increase tumor surveillance (38). It is therefore controversial whether differentiated DCs are also capable of producing immunosuppressive cytokines. Recent work, which applied lymphatic-derived TEVs from melanoma patients, has further supported the theory that DC maturation is targeted by TEV contents. Proteomic profiling of vesicles revealed that this effect might be linked to S100A9, which had been previously described to suppress DC differentiation (39).

The above described findings support the conclusion that the differentiation phase of DCs is particularly susceptible to interference by TEVs. However, when differentiated DCs are incubated *in vitro* with TEVs, some groups were able to use these DCs as a potent DC vaccine against the tumor after expansion in cell culture, underlining the role of TEVs in antigen presentation and induction of the adaptive immune response (40). This provides some evidence that at least a certain subset of TEVs might be part of a tightly regulated cellular anti-tumor signal, but also allows the hypothesis that fully differentiated DCs are less prone to dysregulation by TEVs than their precursors (41). In a pioneering trial applying human material, it was shown that differentiated DCs pulsed with autologous TEVs isolated from patient ascites can be used for inducing tumor-specific CTL, in some cases even expanding restricted T cell repertoires (9). This was the foundation for further TEV/DC-based vaccine trials. Subsequently, antigen transfer from TEVs to DCs has been well characterized. DCs have the capacity to readily internalize TEVs and process their associated antigens in an MHC-dependent manner (28, 42, 43). The uptake of TEVs into DCs has been shown to be mediated by LFA-1/CD54 and C-type lectin/C-type lectin receptor interactions and is dependent on the actin cytoskeleton (40). After internalization, TEV contents are predominantly recruited to MHCII loading compartments (41). Interestingly, TEVs with luminally loaded antigens induce stronger class I MHC responses than TEVs with antigens located at the outside of the vesicle membrane (44). Moreover, TEVs seem to be the most efficient route of presentation for some tumor antigens. Using MUC1, a well-known tumor antigen, Rughetti et al. were able to demonstrate that TEV-associated MUC1 is translocated from endolysosomes to MHCII loading compartments in DCs. This was however not the case when soluble MUC1 was used (45). TEVs also have the capacity to reprogram the uptaking DC by inducing reactive oxygen species (ROS) and alkalinization in the phagosomal compartment, which enhances antigen processing (46). Apart from this, TEV internalization by DCs was also described to result in an increase in expression of the costimulatory receptors CD40, CD80, and CD54 on the DCs (40).

However, it is probable that not all types of TEVs interact with DCs efficiently. For example, a recent study examined glycocalyxes on TEVs, especially ligands of sialic acid binding Ig-like lectin (Siglec) receptors, to determine the extent to which they mediate internalization (47). Indeed, the authors found evidence that these glycocalyx components directly affect TEV-DC interaction and can be experimentally modified to alter the uptake of TEVs by DCs. This finding could explain differences in DC pulsing efficacy, while also allowing for positive selection or artificial generation of TEV subtypes that are well-suited for immunotherapy.

There is also evidence that certain therapeutic interventions shift the payload of TEVs toward a phenotype that favors anti-tumor immune responses. For example, double-stranded DNA (dsDNA) is shed in TEVs (48, 49), but is markedly increased after radiotherapy (RT). This RT-induced dsDNA can be delivered to DCs by TEVs, increasing the production of type 1 interferon to promote the survival of tumor-bearing mice (50). Another study reported that treatment of tumor cells with IFN-γ results in the release of TEVs which have the capacity to induce IL-12 secretion in cultured DCs, thus promoting tumor surveillance (38). This is particularly interesting as this effect was attributed to TEV-associated HSP72, which has been described by other groups as an inducer of pro-tumoral phenotypes in DCs (37). Moreover, others have argued that IFN-γ upregulates the expression of TEV-associated PD-L1, thus inhibiting CTL (22). Further studies to assess how cancer therapy potentially alters the TEV profile toward immune stimulation or suppression are clearly needed as such approaches owe a potential for biomarkers for therapy response.

Taking all these considerations together, the existing evidence points toward several pivotal effects of TEVs on T cells and dendritic cells. First, TEVs are capable of interfering with DC differentiation. Thereby, they inhibit the recognition of tumor-derived antigens and reduce T cell responses in the tumor microenvironment. This is further augmented by activation of regulatory T cells and targeting of CD4^+^/CD8^+^ T cells, in which apoptosis is induced. The potency of TEVs is probably influenced by three factors. First, TEVs circulate within the bloodstream and can therefore reach distant sites of metastasis, where local immunosuppression can contribute to pre-metastatic niche formation (51). Second, a single tumor cell can release thousands of extracellular vesicles during its lifespan and upon undergoing apoptosis. Thereby, the magnitude by which tumor-promoting ligands such as PD-L1 interact with a recipient immune cell is markedly increased compared to single tumor cells (23). Thus, TEVs function like an amplifier of the communication between a tumor and the immune cells in its microenvironment. Third, due to their heterogeneity in biophysical properties and cargo, TEVs can target a large variety of different immune cells, which increases the efficacy by exploiting redundant systems to promote aligned effects. Thus, TEVs largely take part in the cellular reprogramming which is induced by malignant tumors during disease progression (52).

However, as outlined above, it has been also recognized that DCs can process TEV-associated antigens to promote T cell responses. This apparently contradictory finding has prompted irritation in the field whether TEVs are “friends or foes” (53). We aim to answer this question by addressing important experimental differences in the underlying studies in *Explanations for the Reported Heterogeneity of TEV Effects* section.

## TEV Effects on the Innate Immunity: Macrophages and Neutrophils

Few groups have focused on deciphering the interaction of TEVs and the innate immune system. Some evidence exists that macrophages are able to capture TEVs derived from apoptosis from circulation in a CD169 dependent process (54). Knockout of CD169 led to an enhanced immune response after immunization with ovalbumin, suggesting that macrophages eliminate TEVs, thus promoting tolerance. On the other hand, the same group demonstrated that in non-tumor bearing mice, injected TEVs suppress the immune response toward the ovalbumin antigen independent of macrophages (54). In an *in vitro* approach, Bardi et al. found that TEVs polarize macrophages toward a mixed phenotype of both M1 and M2 macrophages, thus promoting both inflammation and tissue regeneration (55). This could be explained by previous studies on macrophages in cancer that described both tumor-suppressing and tumor-promoting roles (56, 57). However, the authors interpreted the mixed macrophage polarization induced by TEVs as evidence of a certain flexibility a tumor would need to induce a vast subset of tumorigenic processes.

In concert with macrophages, neutrophils perform important tasks in immune response, based on recent studies defining interactions of neutrophils with TEVs. Leal and colleagues reported that TEVs induce the formation of neutrophil extracellular traps (NET) that promote cancer-related thrombosis (58). TEVs from tumor stem-like cells, as well differentiated tumor cells were described to induce a pro-tumoral phenotype in neutrophils. This occurs, for example, by polarization toward the N2 subpopulation, which induces production of several factors that promote angiogenesis, invasion and metastasis (59–62). This effect might be due to the uptake of vesicular RNA that exerts signaling functions or the uptake of circulating oncogenic DNA (63). These findings underline the potential of deciphering TEV-based mechanisms by which neutrophils are altered toward a phenotype that promotes tumor survival and progression.

## A Perspective on TEVs in Immunotherapy and Bioengineering

Three leading hypotheses have made TEVs attractive for immunotherapeutic research. First, their potential roles in antigen presentation, possibly owing the potential of an anti-tumor vaccination-like approach, second, their immunosuppressive abilities and, third, their internalization in living cells, making them potential biological vehicles of artificially engineered therapeutic components. Interestingly, as outlined above, immunotherapeutic effects have been described when isolated TEVs are not injected separately into an animal, but are used to educate dendritic cells which are then administered (41). This DC vaccine might be more desirable in terms of therapeutic application compared to the injection of directly tumor-derived material. This approach has been used by several groups to treat cancer in animal models and evaluated in clinical trials that are currently registered using tumor-sensitized autologous dendritic cells or DC-derived EVs (64, 65). However, it remains unknown which tumor antigen source would be most effective for the pulsing of dendritic cells, since it has been reported that certain isolated tumor proteins would be more beneficial than whole tumor cell lysates (66). Gu and colleagues compared tumor lysates and TEVs in a murine model of DC vaccine, finding that the pre-incubation with TEVs is more efficient in terms of tumor growth restriction and survival (41). Furthermore, TEVs could be engineered to have increased MHC expression on the surface of TEVs, and thus enhance the efficacy of DC sensitization (67).

As the field is still at the beginning of exploiting the benefits of EVs for therapeutic purposes, research effort is needed to understand how therapeutic EVs, including TEVs, influence the tumor progression *in vivo*. We have learned from clinical trials that immunomodulatory EVs derived from mesenchymal stromal cells (MSCs) are sufficient to treat steroid-refractory human Graft-versus-Host-Disease (68). However, the experiences with MSC-EVs also demonstrate that various subsets of EVs differ in their immunomodulatory capacities (69). Taking into account the contradictory functional results from studies on TEVs, translation to the clinics could potentially be aggravated by the lack of techniques which are suitable to reliably predict therapeutic efficiency. Therefore, standardized assays, such as those recently proposed by Kordelas et al. (69), are needed in the field to determine the immunomodulatory properties of EV preparations prior to administration. Likewise, the same group has reported that recipients vary in MSC-EV responsiveness which complicates EV therapy (69). Regarding TEVs, this heterogeneity has not been widely addressed in animal models and requires further study. It was recently reported that an *in vitro* assay based on a co-culture of TEVs with splenocytes from an experimental model of murine encephalomyelitis detects TEV-mediated activity that distinguishes MSC-EVs from TEVs based on the expression pattern on targeted splenocytes (70). Further development and validation of similar approaches will likely lead to a platform that has the potential for pre-clinical testing and enhance patient safety, which would be a major step toward the first in-human trials based on TEVs.

Engineered EVs are often investigated with a focus on targeting specific tissues or tumors, respectively. Biodistribution analyses have revealed that EVs are rapidly recruited to tumors after intravenous injection, possibly due to the increased vascularization (71). However, the transport of EVs within the bloodstream to tumor sites would still be unspecific. As integrins have been demonstrated to play a pivotal role in organotropic delivery of EVs (72), this observation could support the loading of specific integrins onto EVs to target the tumor bed. Moreover, exosome-mimetic nanoplatforms (EMN) are potentially useful for the generation of vesicles from cultured tumor cells that are customized to contain specific integrins with a therapeutic payload (73). Such tumor cell-derived EMN share relevant biological properties with “natural” TEVs, but are easier to obtain in therapeutically applicable concentrations. Moreover, the patient safety concerns regarding tumor cell-derived EMN could be addressed by using non-tumor cell lines that retain targeting specificity *in vivo*. For example, Jang et al. reported that monocytes and macrophages can be used for EMN production and that these EMN can be recruited to tumor sites (74), a promising approach avoiding the use of tumor cells.

## Explanations for the Reported Heterogeneity of TEV Effects

**Figure 1:**
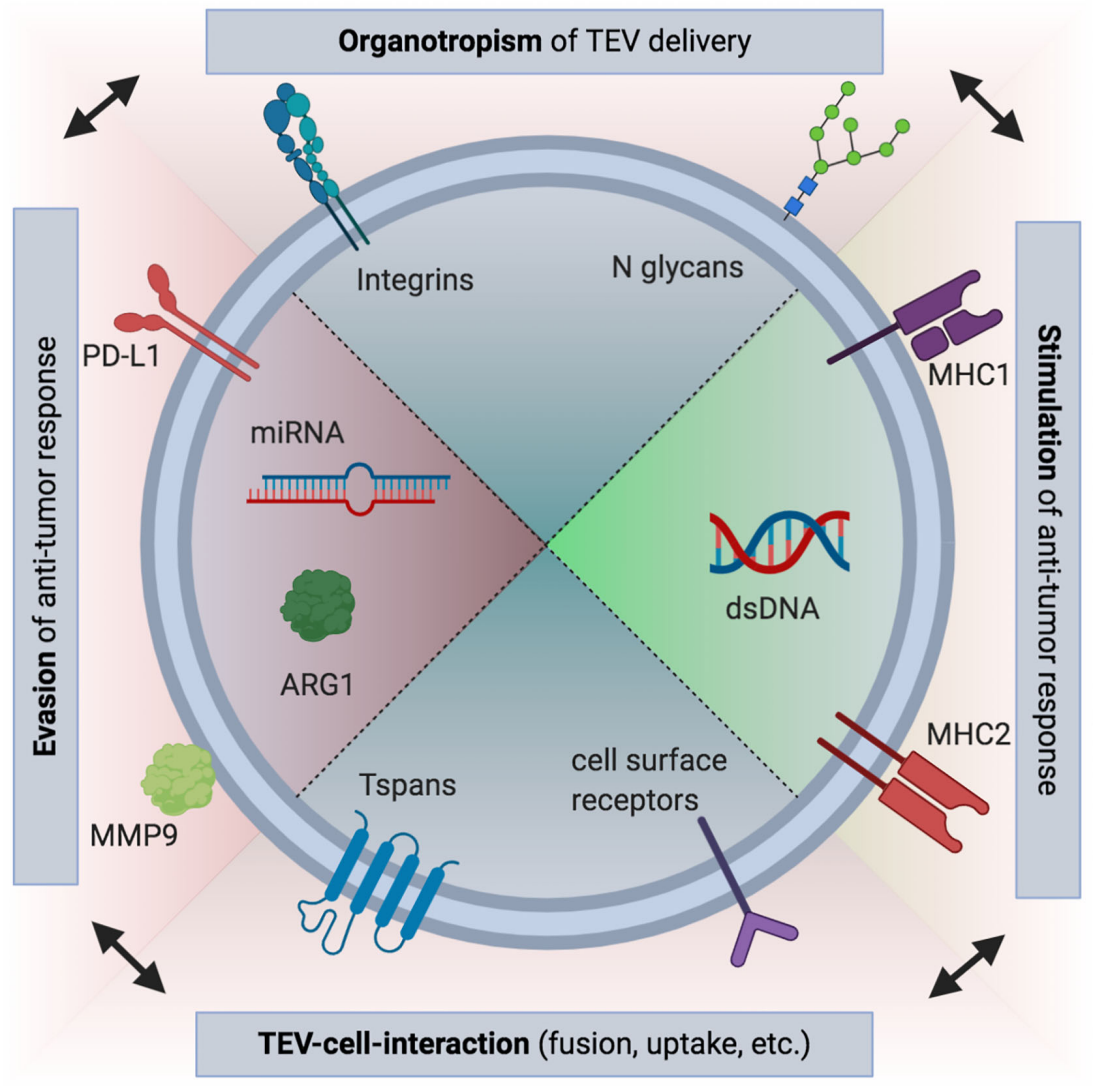
Overview of selected immune-modulatory tumor-derived extracellular vesicle (TEV) cargo. Both immunosuppressive and immune-stimulating roles have been proposed for TEVs. These effects can be mediated by receptors bound to the surface of TEVs, such as PD-L1, or by nucleic acid or protein contents (e.g., arginin-1, ARG1) encapsulated in the interior of the vesicles (21, 22, 25). However, if uptake occurs, interaction of vesicular and cellular receptors is probably needed (75). Tetraspanins (Tspans) such as CD9, CD63, and CD81 are frequently used for characterization of EVs and are likely involved in fusion of TEVs and recipient cells, similar to what has been described for some viruses (76, 77). While also promoting TEV uptake, integrins and other adhesion molecules are responsible for tissue-specific binding of TEVs (78). Thus, immune responses can be modified by TEVs not only globally, but also influence local local responses. This may promote pre-metastatic niche formation (51). Recently, a role in immune cell targeting has been also revealed for N glycans (47). (Figure created using BioRender.com).

**Figure 2:**
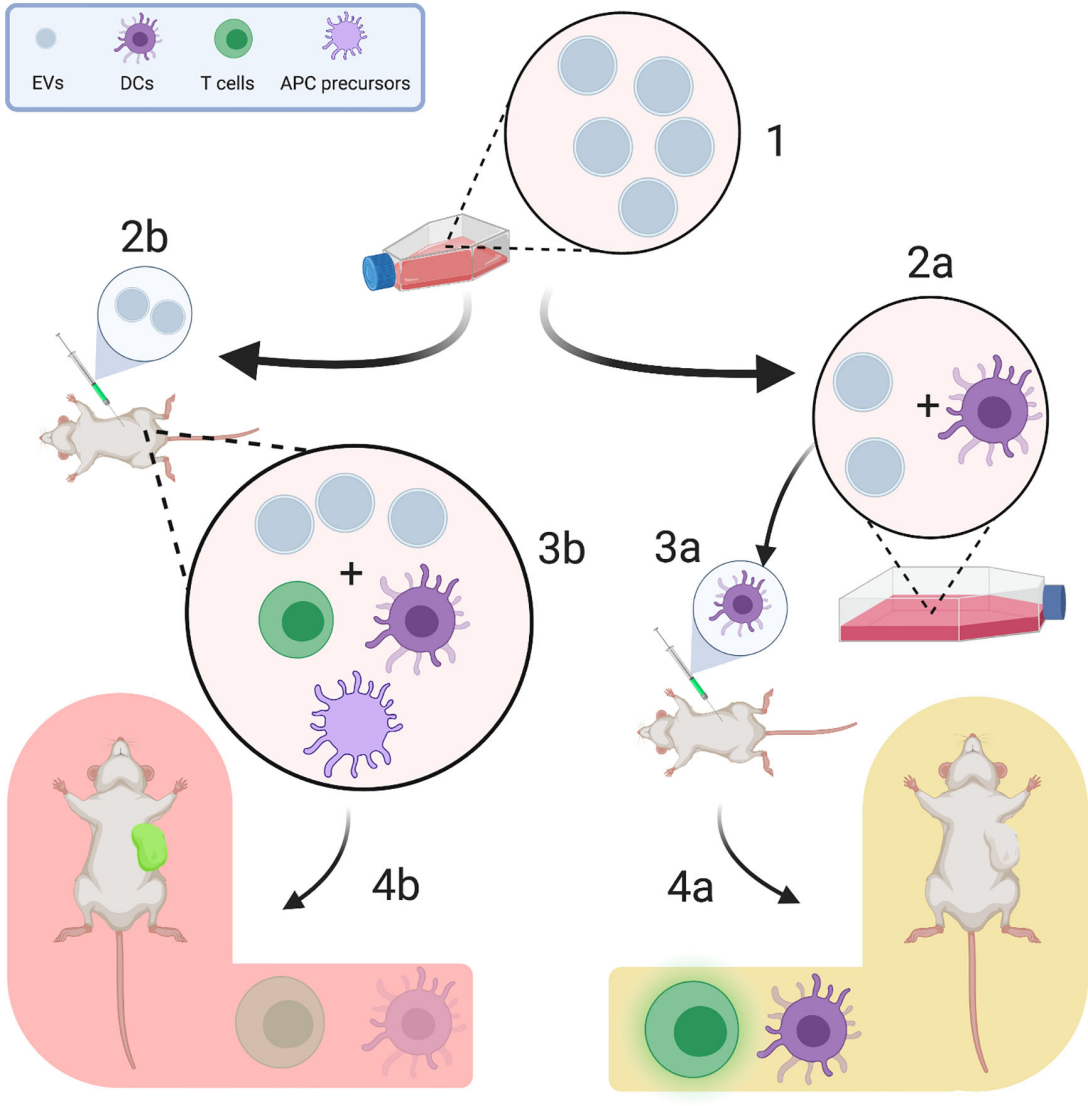
Experimental design influences the heterogeneity of reported tumor-derived extracellular vesicle (TEV) effects. TEVs are regularly harvested from the supernatants of tumor cell lines (*1*). They can be either used for *in vitro* pulsing of immune cells (e.g., differentiated dendritic cells, *2a*) or for direct injection *(2b)*. In pulsing experiments, TEV-associated antigens are presented to dendritic cells, which are then injected into an animal and induce strong T cell responses *(3a)* (41). This promotes tumor growth restriction and increases survival of tumor-bearing mice *(4a)*. On the other hand, when TEVs are injected separately, they interact with a plenitude of other immune cells, such as regulatory T cells, CD34^+^ dendritic cell precursors and myeloid derived suppressor cells *(3b)*, which are commonly activated by TEVs to enable immune evasion (18, 36). This, in turn, reduces the tumor-specific T cell response and enhances tumor progression *(4b)*. (Figure created using BioRender.com).

One of the most enigmatic questions in the study of TEVs remains why TEVs have immunosuppressive as well as immune-stimulatory roles (**Figures 1**, **2**, **Tables 1**, **2**). As outlined above, *in vivo*, TEVs may suppress functionality of dendritic cells and CD4^+^ T cells, and increase activation of Tregs and myeloid-derived suppressor cells (81). However, one could hypothesize that the antigen-presenting component of some TEVs might predominate under certain *in vitro* conditions when the suppressive effect by impaired antigen-presenting cells and supporter cells is lacking. Still, this would not explain why *in vivo* DC vaccines are quite effective when TEVs are used for antigen delivery (9, 41). As a consequence, neither isolated TEVs + effector cells alone nor isolated TEV-pulsed DCs shift the phenotype toward immunosuppression. Of note, the major difference between studies using pulsed DCs vs. direct injection of TEVs in a living animal, is that there is an *in vitro* incubation step of TEVs directly with differentiated DCs, suggesting the following two hypotheses:

- In living organisms, TEVs might strongly influence the differentiation phases of immune cells. This effect has been well described for DCs (36). Rather than interfering with fully differentiated immune cells, TEVs might potently suppress the differentiation of precursor cells.
- In immune-competent animal models as well as in human cancer, TEVs may interact directly with Tregs (activation) (18, 30), CD4^+^ T cells (apoptosis induction) (20), and myeloid-derived suppressor cells (activation) (81). The direct interaction is underlined by the finding that TEV-pulsed dendritic cells do not induce these processes when injected into an animal.

**Table 1:** Tumor-derived extracellular vesicle (TEV)-associated effects on immune cells promoting immune evasion and tumor progression.

Cancer type	Material/cells	Identified cargo	Model	Proposed mode of action	Reference
**Mammary carcinoma**	4T1	PD-L1	*In vitro; in vivo*	Inhibition of DC/Th1 function and differentiation, enhancement of tolerance (Treg induction), partly due to PD-L1	(18)
		MMP9	*In vitro*	Elicitation of pro-inflammatory phenotypes in macrophages ➔ tumor progression	(79)
			*In vitro*	Rab27a-dependent MMP9 secretion independently of TEVs, but may associate with TEV surface	(80)
	TSA	–	*In vitro; in vivo*	Inhibition of bone marrow-derived precursor cell differentiation; induction of IL-6	(36)
	MDA-MB-231	–	*In vitro*	Inhibition of monocyte differentiation into DC	(36)
**Melanoma**	B16-F10	HSP72/105	*In vitro; in vivo*	HSP72/105 induces IL-6 secretion in DCs, dependent on TLR2/4 and STAT3	(37)
		miRNA	*In vitro; in vivo*	miRNA induces apoptosis of CD4+ T cells	(20)
	+ patient material	PD-L1	*In vitro; in vivo*	PD-1-dependent inhibition of CTL proliferation	(22)
	Human melanoma	18 immuno-suppressive proteins	*In vitro*	Direct delivery of inhibitory proteins	(39)
**Prostate carcinoma**	TRAMP-C2	PD-L1	*In vitro; in vivo*	Promotion of tumor growth/progression; suppression of T cell activity	(23)
**Renal cell carcinoma**	RenCa	PD-L1	*In vitro; in vivo*	DC pulsing with TEV enables potent DC vaccine	(41)
		HSP70	*In vitro*	Induction of myeloid-derived suppressor cells, dependent on TLR2/STAT3	(81)
**Lymphoma**	EL4	–	*In vitro; in vivo*	Macrophages eliminate antigen-carrying TEVs to promote tumor tolerance	(54)
**Ovarian cancer**	OvCa + patient material	ARG-1	*In vitro; in vivo*	Delivery of metabolic checkpoint modulators that decrease proliferation of T cells	(25)
	Patient material	FasL	*In vitro*	T cell apoptosis and loss of CD3 zeta chain expression	(82)
**Head and neck cancer**	PCI-13 + patient material	FasL	*In vitro*	Apoptosis induction in activated T cells	(83)
**Acute myeloid leukemia**	Patient material	TGF-β	*In vitro*	Suppression of natural killer cell function	(84)
**Colon carcinoma**	CT26	HSP72	*In vitro; in vivo*	STAT3-mediated activation of myeloid-derived suppressor cells	(85)

**Table 2:** Tumor-derived extracellular vesicle (TEV)-associated effects on immune cells promoting tumor surveillance and immune response.

Cancer type	Material/cells	Identified cargo	Model	Proposed mode of action	Reference
**Mammary carcinoma**	4T1	HSP72	*In vitro*	HSP72 induces IL-12 release by DCs	(38)
	TSA	dsDNA	*In vitro; in vivo*	Delivery of dsDNA to DCs after radiotherapy	(50)
**Melanoma**	B16-F10	–	*In vitro*	Macrophage polarization toward mixed, pro-tumoral phenotypes	(55)
	B16-F1	–	*In vivo*	Protection against tumor growth after TEV injection	(86)
	Human melanoma	Matr1 antigen	*In vitro*	Induction of DCs and antigen-specific CTL	(9)
**Renal cell carcinoma**	RCC	–	*In vitro; in vivo*	Stimulation of CTL through FasL/Fas signaling	(26)

Taking *(a)* and *(b)* together, one can conclude that at least some subtypes of TEVs have the capacity to present tumor antigens to CD8^+^ T cells and DCs. However, in a living organism, interactions are much more complex. Direct effects of TEVs on T-suppressor cells have to be considered where TEVs themselves are involved in a system of linking regulatory immune pathways. Moreover, naïve immune cells might react substantially differently to TEVs than exhausted T cells or DCs in the tumor microenvironment. Therefore, one explanation for the efficacy of DC vaccines could be that the injected DCs are not excessively reprogrammed toward a pro-tumoral phenotype, unlike the endogenous DCs. Once more, this implicates distinct functions of TEVs under various conditions.

Moreover, the role of TEVs has to be integrated into the complex network of the tumor secretome. This includes soluble factors that are not released in vesicles, but might – due to their biochemical properties – adhere to the surface of vesicles or might as well remain completely unassociated with TEVs (87). Therefore, it might be even harder than expected to match the biological origin of TEVs with their function, and to establish the effect of TEVs that could be over- or underestimated. For example, Madera and colleagues demonstrated that the macrophage-related effects of breast cancer cell conditioned media are partly, but not exclusively, due to TEVs (79). These observations underscore the importance of detailed attention to controls in functional studies, such as the methodology for the collection of conditioned media depleted of TEVs.

Apart from these considerations, another explanation could be the heterogeneity of EVs themselves. However, the question of which subtype of TEVs is most likely involved in the various processes of immunomodulation has not been widely assessed, as there are hardly any well-established markers that could easily distinguish exosomes from microvesicles or apoptotic bodies, respectively. Nevertheless, some groups have targeted this question, including further characterization of the immune-modulatory TEVs. Muhsin-Sharafaldine and colleagues observed that the antigen-presenting role of TEVs is mostly mediated by apoptotic vesicles, as tumor antigens were only detected in the “apoptotic vesicle-enriched fraction” (86). Subsequently, when mice were first immunized by B16-F1 cell line-derived EVs and then subject to B16 melanoma implantation, the group of animals which had been “vaccinated” with apoptotic vesicles were protected for longer from the tumor than animals that were administered exosomes and microvesicles. However, apoptotic bodies were identified as vesicles larger than 200 nm, although it is also accepted that some apoptotic bodies are actually as small as 100 nm (88). Of note, exosomes were – among other markers – characterized by the presence of histones. It is currently extremely controversial whether histone proteins are presents in EVs at all, and the characterization of exosomes by histone payload is therefore not recommended at the moment (7). Regardless, the hypothesis that apoptotic-derived vesicles carry at least a larger amount of tumor antigens than exosomes is consistent with the understanding of exosome biogenesis as a tightly controlled process versus apoptotic vesicle formation as a part of “cell waste” disposal. Moreover, the immunogenicity of apoptotic tumor cells has been well described (89, 90). Indeed, apoptotic vesicles could induce comparably strong anti-tumor immune responses, as the variety of genetic material and tumor-derived protein content is probably the largest among the vesicle types.

Recently, new light was shed on the functional differences between the several types of TEVs. In a study published by Temoche-Diaz et al., two subtypes of small TEVs from a breast cancer cell line were successfully separated by high-resolution density gradient fractionation and then further characterized (91). These authors demonstrated that these two subtypes of small TEVs exploit distinct mechanisms of RNA loading and recruit miRNA in a selective way. As gene ontology analysis revealed different subcellular origins of these vesicles, corresponding to endosomes and the plasma membrane, it can be hypothesized that the rough EV pellet typically obtained by ultracentrifugation contains at least two biologically different populations of TEVs with possibly distinct functions. Given the fact that these subtypes were shown to differ in terms of their density, even minimal differences in ultracentrifugation parameters such as k-factor, speed, time, and rotor (swing-out vs. fixed angle) or tube type used might predominantly enrich one of these subtypes. As a remarkable number of publications do not contain any characterization of the proposed EVs or only provide evidence that significantly falls below the experimental guidelines of the ISEV (8), findings should also be interpreted with a view to the utilized TEV isolation and characterization techniques.

Finally, it is notable that tumor cell lines differ substantially in terms of their invasiveness, apoptosis rate and metastatic potential. All of these factors are probably reflected in the formation of TEVs and therefore contribute to the heterogeneity of reported TEV effects, as well *in vitro* as *in vivo*. Of note, it was demonstrated that cancer cell lines differ in the expression of Rab27a, one of the known regulators of EV release (80). Another example is Rab7, which regulates MVB transport toward the plasma membrane and is differentially expressed in ovarian/peritoneal serous carcinoma and malignant peritoneal mesothelioma (92). Differences in the abundance of SNARE proteins, which mediate the fusion of MVBs and the plasma membrane, have been described for various types of hematopoietic and lymphoid neoplasia (93). Apart from release-associated proteins and enzymes, molecules that exert direct signaling functions are differentially expressed in various tumors. For example, PD-L1 is a prominent example that has been linked to TEV-induced immunosuppression and is a promising clinical outcomes marker following immunotherapy (23, 94).

Taking all these considerations together, several lessons have to be learned from functional animal experiments studying the effect of TEVs on the immune response. Obviously, findings from *in vivo* and *in vitro* experiments differ notably (**Figure 2**). Although comparative studies are largely lacking, preliminary evidence suggests that experimental differences in *in vitro* vs. *in vivo* trials account for a proportion of the reported heterogeneity of TEV effects (95). Cell culture experiments are especially useful if interactions between well-defined components are studied. These components should be arbitrarily manipulatable by the researcher. However, this is not the case for functional TEV-immunity experiments, as two complex systems – the vesicle population and the immune system – are studied, which are necessarily oversimplified in this experimental setting. Therefore, cell culture trials of immune cell interactions with TEVs resemble a burning lens – they apply a strong focus on specific details but leave the rest blurred out. This is underlined by two major observations that have been outlined above:

First, mouse models have shown that many different immune cells and precursors are influenced by TEVs. The immune cells, in turn, influence each other’s functionality but also the tumor microenvironment. It is therefore conceivable that the TEV composition changes after targeting of the tumor by immune cells. Moreover, tumor progression leads to a substantial reprogramming of cellular functions, thus, in later tumor stages, e.g., the antigen-presenting role of TEVs could be heavily impaired. Certain TEV types themselves could contribute to this process (96). These complex biological crosslinks are impossible to mimic in a cell culture experiment which cannot simulate reciprocal interactions.

Second, any experimental TEV population is highly heterogeneous. Researchers are confronted with two sides of the same coin: on one hand, technical purification of TEVs is necessary for pulsing immune cells *in vitro*. On the other hand, extreme purification can also mean a significant loss of EV subpopulations. Moreover, the purity of EV preparations is an issue that has led to misinterpretations in terms of cargo (7). Thus, an impact on experimental outcomes cannot be excluded.

From a technical perspective, animal models owe the unique potential to address currently understudied questions more rigorously. For instance, *in vivo* TEV tracking could provide insight into details on the kinetics of TEV-immune cell interactions. Lipid dyes, including, e.g., CFSE, PKH26/67 and DiR, have been applied in functional mouse models of tumor progression (97), although there are remaining concerns regarding their lack of staining specificity. A main issue is the labelling of non-EV-lipid particles, such as lipoproteins, which surpass EVs in terms of abundancy. Thus, results may not be exclusively attributed to EVs. A comprehensive review of fluorescent dyes for EV staining has been recently published elsewhere (98). The problem of TEV pre-staining has been addressed by multiple groups, who applied transfection of tumor cells with a fluorescent reporter such as green fluorescent protein (GFP) under the regulation of known EV associated proteins like the tetraspanins CD9, CD63, and CD81 (99, 100). Combined with recent advances in high resolution imaging and nanoscale flow cytometry, TEV release and trafficking can be further addressed (101, 102). Another important aspect is the mode of administration of TEVs in laboratory animals. If non-tumor bearing animals are used, immunological effects might differ notably from the effects observed in tumor-bearing animals, in which TEVs are constitutively released. Additionally, the optimal amount of TEVs injected into animals as well as the administration route will likely have an impact on functional results (103). To overcome the problems of TEV pre-isolation, staining, and administration, the above described expression of fluorescent or bioluminescent TEV fusion protein reporters could be further investigated in murine tumors. Bridging techniques, such as a recently reported spheroid co-culture model of PBMCs and tumor cells stably releasing GFP-tagged TEVs (33), offer a valuable chance to assess functional hypotheses before planning such animal trials.

## Concluding Remarks

During the past decade, our understanding of tumor-derived EVs has grown tremendously. However, translation into clinical research has been delayed by contradictory findings and the technical complexity of EV studies. As substantial progress has been recently made especially in the field of nanotechnology, further advances in nanoscale bioimaging might ultimately enable rigorous animal trials, which could elucidate the enigmatic dichotomy of TEV functions in cancer immunity. This, in turn, will lead to a more detailed understanding of the mechanisms by which TEVs alter immune responses toward a pro-tumoral phenotype, owing the potential for therapeutic manipulation. We have argued that animal models can much better account for the heterogeneity of both the TEV populations and immune cell functions. They also accurately display physiological phenomena such as immune cell exhaustion, tumor apoptosis and invasion. Moreover, they owe the potential to study alterations of the TEV profile in different phases of tumor development and progression. Given these premises, future research efforts should focus on pathophysiological models in the tumor-bearing living animal rather than characterization of TEV-single-cell-interactions. Multiparametric readouts, using techniques that combine high-resolution imaging and biochemical characterization, e.g., *via* nanoscale flow cytometry, are valuable to cover a broad spectrum of immune cell interactions.

Moreover, the antigen-carrying functions of TEVs and their role in dendritic cell vaccines will likely cause further research enthusiasm in clinical oncology. However, to fully exploit the undoubted potential of TEVs as immunotherapeutic agents, standardized assays will be necessary to evaluate the biological activity of TEV preparations. Ultimately, ongoing efforts might open the door for TEVs as a novel component of personalized tumor therapy, either as a novel treatment or a therapeutic target.

## Author Contributions

MD wrote the review with editorial advice from BT and BE. All authors contributed to the article and approved the submitted version.

## Funding

MD received funding support by the Else Kroener Fresenius-Stiftung foundation and the German Academic Scholarship Foundation (“Studienstiftung des deutschen Volkes”). BT is supported by Stiftung Universitätsmedizin Essen. Additional support (BE) was provided by the National Institutes of Health (R01 GM140137) and the Academic Senate of UC San Diego and the Department of Surgery.

## Conflict of Interest

The authors declare that the research was conducted in the absence of any commercial or financial relationships that could be construed as a potential conflict of interest.
